# Combination of *BCL11A* siRNA with vincristine increases the apoptosis of SUDHL6 cells

**DOI:** 10.1186/2047-783X-19-34

**Published:** 2014-06-24

**Authors:** Dongmei He, Hong Wu, Li Ding, Yangqiu Li

**Affiliations:** 1 Institute of Hematology, Medical College, Jinan University, No. 601, West Huangpu Road, Guangzhou, Tianhe District 510632, PR China; 2 Key Laboratory for Regenerative Medicine of Ministry of Education, Jinan University, No. 601, West Huangpu Road, Guangzhou, Tianhe District 510632, PR China

**Keywords:** BCL11A, small interfering RNA, vincristine, SUDHL6 cells, apoptosis

## Abstract

**Background:**

B cell chronic lymphocytic leukemia/lymphoma 11 A (BCL11A) is associated with human B cell malignancy initiation. Our previous study has shown that downregulation of *BCL11A* mRNA by small interfering RNA (siRNA) is capable of inducing apoptosis in the SUDHL6 cell line. To further explore the effects of *BCL11A* siRNA on the enhanced cytotoxicity of a chemotherapeutic drug, we investigated the effects of *BCL11A* siRNA combined with vincristine (VCR) on SUDHL6 cell proliferation and apoptosis.

**Methods:**

Chemically synthesized *BCL11A* siRNA was transfected into SUDHL6 cells using the HiPerFect Transfection Reagent in combination with VCR. Cell proliferation was measured by the CCK8 assay. The morphology of apoptotic cells was observed with Hoechst 33258 staining. The rate of cell apoptosis was determined by annexin V-fluorescein isothiocyanate/propidium iodide double staining using fluorescence-activated cell sorting (FACS) analysis.

**Results:**

After *BCL11A* siRNA plus VCR treatment, cell proliferation was significantly decreased in comparison with VCR or *BCL11A* siRNA treatment alone and negative control siRNA plus VCR treatment (*P* <0.05). The apoptotic rate of *BCL11A* siRNA plus VCR treated cells was significantly increased compared with *BCL11A* siRNA and VCR treatment alone and negative control siRNA plus VCR treatment (*P* <0.05).

**Conclusions:**

The combination of *BCL11A* siRNA and VCR increases apoptosis in SUDHL6 cells. Our study implies that *BCL11A* siRNA in combination with VCR may be a useful approach for improving effective treatment for B cell lymphoma.

## Background

Diffuse large B cell lymphoma (DLBCL) is the most common type of non-Hodgkin lymphoma (NHL) [1,2]. DLBCL is a heterogeneous disease in its morphology, immunophenotype, and biological behavior, and it includes subtypes with diverse origins and gene expression profiles [1,3]. Current treatments for NHL are not optimally effective and mainly result in relapse and resistance to chemotherapy [4,5]. Therefore, further investigation of specific DLBCL biomarkers, development of more targeted treatments, and improvement of the effects of treatment are indispensable for significantly increasing the survival of patients.

B cell chronic lymphocytic leukemia/lymphoma 11 (*BCL11*) gene family members, including the BCL11A and BCL11B, have been identified as transcriptional repressors, which are essential for lymphoid development [6-8]. The *BCL11B* gene, which is related to malignant T cell transformation, plays a crucial role in the development, proliferation, differentiation and subsequent survival of T cells [9]. *BCL11A* has been identified on human chromosome 2p16.1 (previously mapped at 2p13) where chromosomal abnormalities are associated with human lymphoma [10,11]. Recently, Yin *et al*. suggested that *BCL11A* acts as an oncogene and may contribute to leukemogenesis in certain groups of AML patients [12]. BCL11A overexpression is primarily found in B cell lymphoma and B cell leukemia [11,13-16]. We and others have demonstrated the essential role of BCL11A in the proliferation and survival of B cells [8,17]. Our previous study has shown that downregulation of *BCL11A* mRNA by small interfering RNA (siRNA) is capable of inducing apoptosis in B lymphoma cell lines (SUDHL6 and EB1) [17]. Gene expression profiling revealed that various genes related to apoptosis and proliferation are altered during *BCL11A* siRNA-mediated SUDHL6 cell apoptosis (WH and Gao Yangjun, unpublished data).

Vincristine (VCR) is a commonly used chemotherapeutic agent for many lymphoid malignancies, including aggressive NHL. Depending on the therapeutic dose, most chemotherapeutic agents have side effects. VCR has additional peripheral neurological side effects such as hearing changes, sensory loss, numbness, and tingling [18]. Serious side effects in response to chemotherapeutic agents led researchers to seek novel anticancer agents with fewer side effects, and these newly explored anticancer agents can be used in combination with commonly used chemotherapeutic agents to reduce serious side effects [19-22]. A recent report suggested a possible synergy between VCR and the amino acid-depleting agent pegylated arginase I (BCT-100) in treating T-ALL in the cancer microenvironment [23].

RNA interference (RNAi)-based therapeutics has emerged for the treatment of various human diseases including cancer [22,24]. Based on the efficacy of *BCL11A* siRNA in inhibiting SUDHL6 cells [17], we hypothesized that *BCL11A* siRNA plus VCR enhances inhibitory activity in SUDHL6 cells. To the best of our knowledge, our findings indicate for the first time that *BCL11A* siRNA increases VCR-induced apoptosis in SUDHL6 cells. Therefore, our study implies that the combination of *BCL11A* siRNA transfection plus VCR is an efficacious therapeutic approach for treating B cell lymphomas that express BCL11A.

## Methods

### Reagents

*BCL11A*-specific siRNA (sense: GAAUCUACUUAGAAAGCGATT and antisense: UCGCUUUCUAAGUAGAUUCTT, Chinese patent number: ZL 2011 1 0301731.6), which targets domains in the third exon of the *BCL11A* gene (ACCESSION NM_022893.3), [EMBL:AJ404611], and its corresponding non-silencing negative control siRNA were designed and synthesized by Shanghai GenePharma Co., Ltd. (Shanghai, China). RPMI 1640 and newborn calf serum were purchased from Gibco (Gibco, Carlsbad CA, USA). VCR was purchased from Shenzhen Main Luck Pharmaceuticals, Inc (Shenzhen, Guangdong, China).

### Cell culture and transfection

The SUDHL6 cell line, which was derived from germinal center B cell-like DLBCL, was kindly provided by Professor Ailin Guo from the Department of Pathology (Cornell University, Ithaca, NY, USA). The cells were cultured in RPMI medium supplemented with 10% heat-inactivated fetal calf serum at 37°C under 5% CO_2_ in a humidified incubator. SUDHL6 cells in the exponential growth phase were grown for 24 hours and then transfected using HiPerFect (Qiagen, Valencia, CA, USA) according to the manufacturer’s protocols. In addition, cells were transfected with negative control siRNA. The total concentration of siRNA applied in every case was maintained constant at 100 nM.

### Assay of cell viability

For the quantitative determination of cellular proliferation and viability, we performed the CCK8 assay. This assay was performed after SUDHL6 cells were transfected with *BCL11A* siRNA in combination with VCR (1 μM) at 24, 48 and 72 h. The cells were washed, counted and seeded at a density of 4 × 10^5^ cells/ml per well in 96-well plates. Six hours later, *BCL11A* siRNA in combination with VCR was added to the cells. At 48 and 72 h after transfection, CCK8 solution was added 4 h before the end of incubation. Cell viability was measured with a spectrophotometer at an absorbance of 450 nm. The inhibition rates of cell growth were calculated according to the following formula: inhibition rate (%) = (1 ‒ mean absorbance of treatment group/mean absorbance of untreatedmentgroup) × 100%.

### Assays of cell apoptosis

Transfected SUDHL6 cells were harvested after treatment. Morphology was determined with Hoechst 33258 following incubation for 72 h. Cells were washed with PBS three times and then stained with 10 μl Hoechst33258 nuclear dye (KeyGEN, Nanjing, China) for 10 min at 37°C. After cells had been washed with PBS three times, images were obtained with a fluorescence microscope (Leica, Germany). Apoptosis assays were performed using annexin V-fluorescein isothiocyanate (annexin V-FITC) and propidium iodide (PI) (BD Pharmingen, San Jose, CA, USA) according to the instructions of the manufacturer. Briefly, cells were centrifuged, washed with cold PBS, and then resuspended in 500 μl binding buffer. FITC-conjugated Annexin V (10 μl) and PI (10 μl) were added to each sample, and the mixture was incubated at 4°C in the dark for 5 min. The cells were then immediately subjected to fluorescence-activated cell sorting (FACS) analysis (BD FACS Calibur, Franklin Lakes, New Jersey, USA). The percentage of early and late apoptotic cells in each group was determined.

### Statistical analysis

Results are shown as the mean ± s.d. Statistical comparisons were made using ANOVA. Differences were deemed significant for a real alpha of 0.05. All statistical analysis was performed with SPSS 13.0.

## Results and discussion

BCL11A is a Krüppel-like transcription factor that is closely related to B cell proliferation and differentiation [6,8]. High expression of BCL11A is thought to possibly be involved in the genesis of B cell neoplasms [11,13-16]. We found that downregulation of *BCL11A* expression by siRNA can inhibit proliferation and induce apoptosis in SUDHL6 cells [17]. Gene expression profiling of *BCL11A* siRNA-treated SUDHL6 cells in our laboratory also highlighted that *BCL11A* might be related to a variety of signaling networks including apoptosis and the cell cycle (WH and Gao Yangjun, unpublished data). In this study, consistent with our previous report, *BCL11A* siRNA inhibited the proliferation and growth of SUDHL6 cells [17]. These results suggest that oncogenic BCL11A may be a rational therapeutic target in B cell lymphoma. Hence, of particular interest is to observe whether *BCL11A* siRNA significantly enhances the therapeutic efficacy of chemotherapeutic drugs in B cell malignancy. Therefore, we attempted to explore the combinatorial effects of *BCL11A* siRNA and a chemotherapeutic agent (VCR).

**Figure 1:**
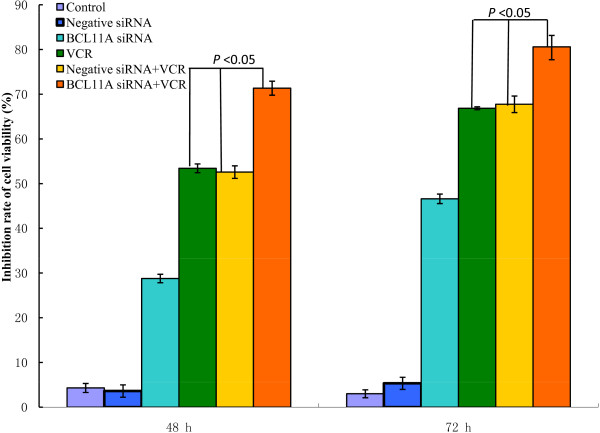
**The rate of inhibition of SUDHL6 cell growth as measured by the CCK8 assay after combined treatment with *BCL11A *siRNA plus vincristine (VCR).** SUDHL6 cell proliferation was determined by the CCK8 assay. The inhibition rates for cell growth were calculated according to the following formula: inhibition rate (%) = (1 - mean absorbance of treatment group/mean absorbance of untreated group) × 100%, and the resulting values were plotted. The results represent mean values from three independent experiments ± s.d.

**Figure 2:**
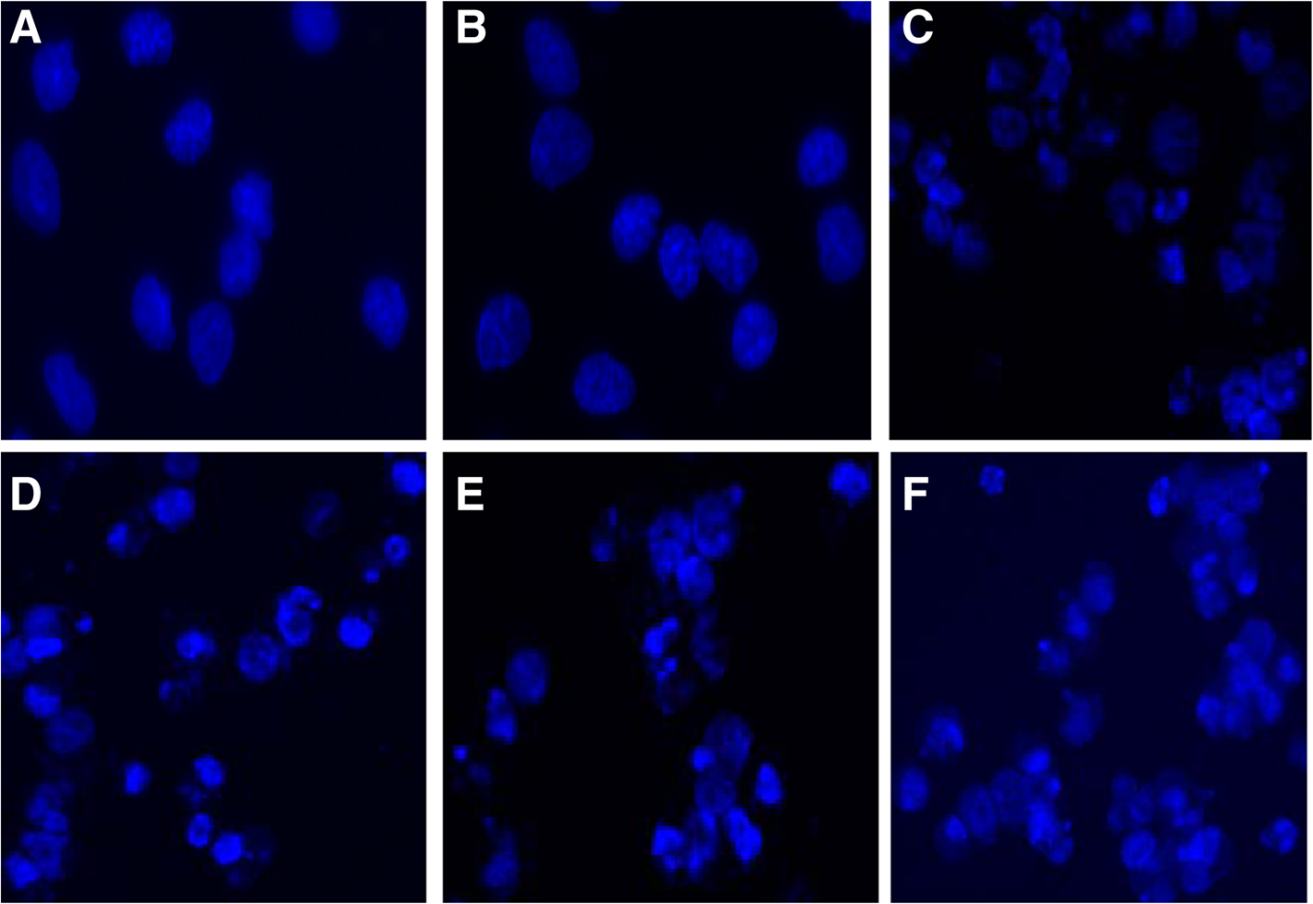
**The morphology effects from the combination of *BCL11A *siRNA plus vincristine in SUDHL6 cells at 72 hours.** The nuclear morphology of cells stained with Hoechst 33258 was analyzed by fluorescence microscopy (400×) at 72 h after transfection. Data are representative microscopic images of three independent experiments. **A)** untreated cells; **B)** negative control siRNA; **C)***BCL11A* siRNA; **D)** vincristine; **E)** negative control siRNA plus vincristine; **F) ***BCL11A* siRNA plus vincristine.

**Figure 3:**
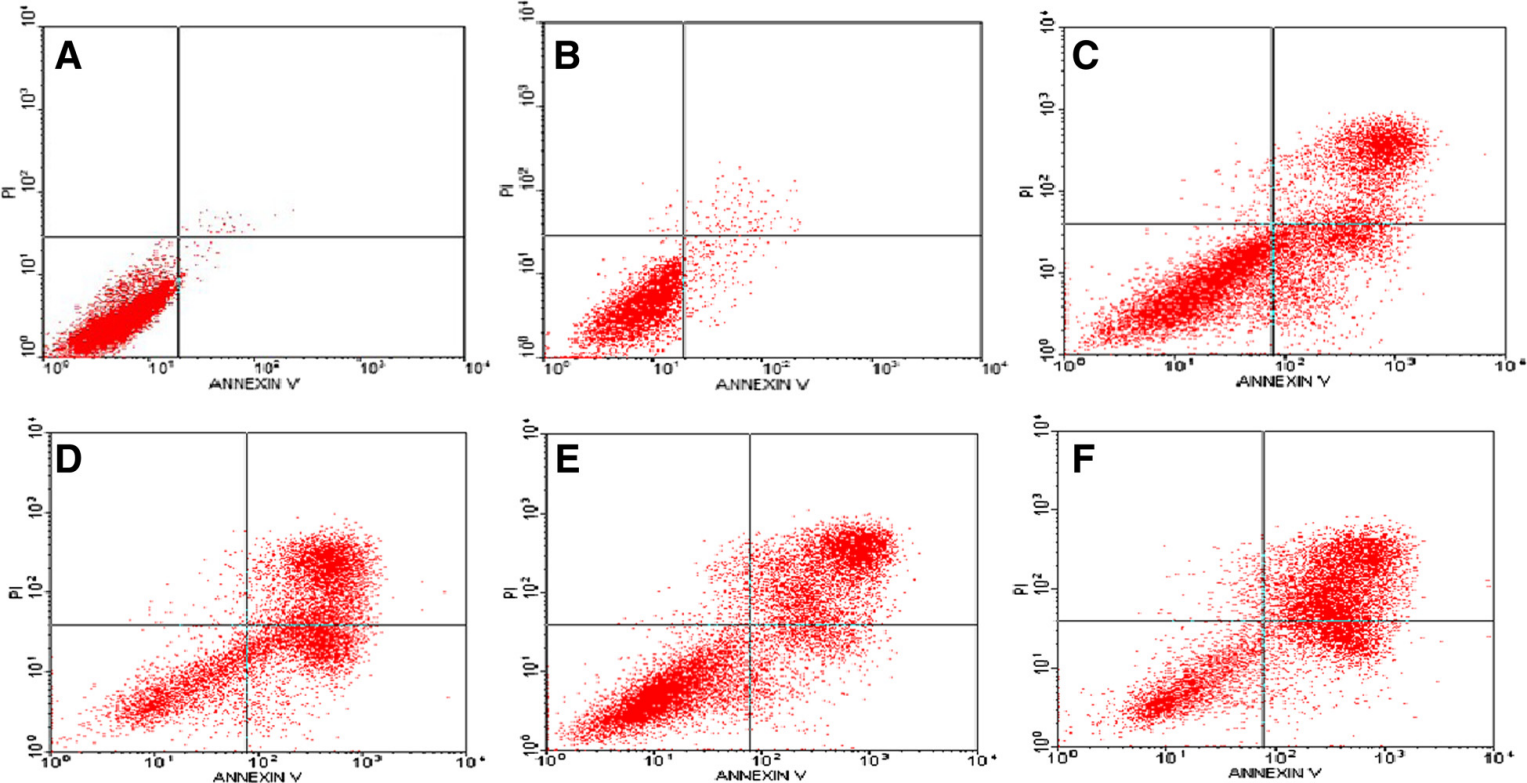
**The rate of apoptosis induced by the combination of *BCL11A *siRNA and vincristine in SUDHL6 cells at 72 hours.** Apoptosis was analyzed by annexin V-FITC/PI staining 72 h after treatment. Data are representative images of three independent experiments. **A)** untreated cells; **B)** negative control siRNA; **C) ***BCL11A* siRNA; **D)** vincristine; **E)** negative control siRNA plus vincristine; **F) ***BCL11A* siRNA plus vincristine.

Interestingly, our results demonstrated that cell viability was more effectively reduced by *BCL11A* siRNA combined with VCR in a time-dependent fashion (*P* <0.05) (Figure 1). Furthermore, in order to identify the mechanism responsible for this decline in cell viability, a morphology assay was used. Our results showed significant apoptotic morphology changes such as chromatin condensation and fragmentation at 72 h after transfection with *BCL11A* siRNA in combination with VCR in SUDHL6 cells (Figure 2). Moreover, there was a large increase in annexin V- FITC/PI double-positive cells at 72 h after transfection with *BCL11A* siRNA in combination with VCR in SUDHL6 cells. The apoptosis rate of *BCL11A* siRNA combined with VCR treatment was (71.46 ± 2.53)%, which was statistically significantly different compared with *BCL11A* siRNA (39.64 ± 5.17)% or VCR (49.73 ± 6.74)% treatment alone (*P* <0.05) (Figure 3). There was no significant difference compared with VCR and the negative control siRNA plus VCR treatment (50.32 ± 6.18)%. Therefore, this study showed that *BCL11A* siRNA in combination with VCR enhances apoptosis, thereby inhibiting the proliferation and growth of SUDHL6 cells. These results indicate that there is a potential therapeutic benefit for the combination of *BCL11A* siRNA and VCR compared with *BCL11A* siRNA or VCR alone.

These results are consistent with a related report that showed that silencing the antiapoptotic protein Bfl-1 by siRNA in DLBCL cell lines induced apoptosis and sensitized those cells to apoptosis induced by chemotherapeutic compounds such as doxorubicin, vincristine, cisplatin and fludarabine [25].

SUDHL6 cells, which were chosen for this study, are characterized by a t(14;18) chromosomal translocation resulting in overexpression of the antiapoptotic protein BCL-2. In SUDHL6 cells transfected with *BCL11A* siRNA, we found that the apoptosis gene *BIM* (BCL2-interacting mediator of cell death) was upregulated, and the anti-apoptosis genes *BCL-2* and *MDM2* (murine double minute 2) were downregulated (WH and Gao Yangjun, unpublished data). A recent study has suggested *BCL11A* deletion causes apoptosis in early B cells *in vivo* and *in vitro* by directly regulating *BCL-2*, *BCL-xL*, *MDM2*, and *MDM4*[8].

Two research groups demonstrated that the small molecule ABT-737, a BCL-2 homology domain 3 mimetic, in combination with VCR increased the apoptotic cell death of leukemia cell lines [26,27]. Our previous studies and other groups have suggested that downregulation of *BCL-2* expression by siRNA increases the sensitivity of human tumor cells to chemotherapeutic drugs [28-30]. MDM2 negatively regulates the activity of the tumor suppressor protein p53, thus having an anti-apoptotic role [31]. MDM2 inhibition by the antagonist nutlin-3 sensitizes neoplasm cells to chemotherapy-induced apoptotic cell death [32,33]. The mechanism of induced apoptosis mediated by VCR is complex and involves protein kinase signaling pathways [34]. Mitochondria also appear to play a key role in this process [35]. Based on these findings, we inferred that the *BCL11A* siRNA plus VCR-induced apoptosis of SUDHL6 cells might be related to the downregulation of BCL-2 and MDM2 and upregulation of BIM*.* However, how *BCL11A* siRNA and VCR are involved in enhancing the apoptotic process by BCL-2, MDM2 and/or BIM needs further investigation.

## Conclusions

We demonstrated that combined treatment with *BCL11A* siRNA and VCR increases the inhibitory effects of *in vitro* cell growth and apoptosis in SUDHL6 cells. Our study indicates that the combination of *BCL11A* siRNA and VCR offers a novel and potential therapeutic strategy for B cell malignancies. However, further investigation of a wide range of B lymphoma cell types and chemotherapeutic drugs and *in vivo* studies confirming the efficacy of this treatment paradigm are necessary.

## Abbreviations

BCL11A: B cell chronic lymphocytic leukemia/lymphoma 11A; BIM: BCL2-interacting mediator of cell death; DLBCL: diffuse large B cell lymphoma; FACS: fluorescence-activated cell sorting; FITC: fluorescein isothiocyanate; GCB: germinal center B cell-like; MDM2: murine double minute 2; NHL: non-Hodgkin lymphoma; PI: propidium iodide; RNAi: RNA interference; siRNA: small interfering RNA; VCR: vincristine.

## Competing interests

None of the authors have any competing interests for the results reported in this study.

## Authors’ contribution

DMH designed and performed experiments, interpreted results and contributed to the writing of the manuscript. HW and LD performed experiments and interpreted results. YQL developed the original concept and designed and contributed to the writing of the manuscript. All authors read and approved the final manuscript.
